# Characterization of a case of follicular lymphoma transformed into B-lymphoblastic leukemia

**DOI:** 10.1186/1755-8166-6-34

**Published:** 2013-08-28

**Authors:** Yi Ning, Aubry Foss, Amy S Kimball, Nicholas Neill, Tricia Matz, Roger Schultz

**Affiliations:** 1 Department of Pathology, Johns Hopkins University, Baltimore, MD, USA; 2 Signature Genomic Laboratories/Perkin Elmer, 2820 N Astor, Spokane, WA, 99207, USA; 3 Greenebaum Cancer Center, University of Maryland School of Medicine, Baltimore, MD, USA

**Keywords:** Follicular lymphoma, Transformation, B-Lymphoblastic leukemia, Translocation, Microarray

## Abstract

Follicular lymphoma (FL) is a common form of non-Hodgkin lymphoma with an ability to transform into a more aggressive disease, albeit infrequently to B-lymphoblastic leukemia/lymphoma. While t(14;18)(q32;q21) has been associated with approximately 90% cases of FL, that alteration alone is insufficient to cause FL and associated mutations are still being elucidated. The transformation of FL to B-lymphoblastic leukemia generally includes the dysregulation of *MYC* gene expression, typically through *IGH* rearrangement. Such cases of “double-hit” leukemia/lymphoma with both *BCL2* and *MYC* translocations warrant further study as they are often not identified early, are associated with a poor prognosis, and are incompletely understood in molecular terms. Here we describe a patient with a diagnosis of FL that transformed to B-lymphoblastic leukemia. Detailed cytogenetic characterization of the transformed specimen using karyotype, fluorescence in situ hybridization, microarray and gene rearrangement analyses revealed a complex karyotype comprised principally of whole chromosome or whole arm copy number gains or losses. Smaller, single-gene copy number alterations identified by microarray were limited in number, but included amplification of a truncated *EP300* gene and alterations in *NEIL1* and *GPHN*. Analyses defined the presence of an *IGH/BCL2* fusion due to a translocation as well as a *MYC/IGH* fusion due to an insertion, with both rearrangements involving the same *IGH* allele. The data illustrate the value in characterizing double-hit lymphoma cases with both traditional and novel technologies in the detailed cytogenetic workup.

## Background

Follicular lymphoma (FL) accounts for approximately 30% of adult non-Hodgkin lymphoma and is generally indolent in its clinical course. A subset of FL cases transform into a more aggressive disease, most often to diffuse large B-cell lymphoma [1]. Transformation to other entities, such as B- acute lymphoblastic leukemia (B-ALL), is less common [1-3]. FL is genetically characterized by the presence of the t(14;18)(q32;q21) chromosomal translocation in approximately 90% of cases [4]. However, that alteration alone is insufficient to cause FL and those additional genomic events specifically leading to disease are still being elucidated. Similarly, those events that mediate transformation of FL to B-ALL remain under investigation, although dysregulation of *MYC* gene expression typically through rearrangement with the immunoglobulin (IG) locus is a common finding. Such cases of “double-hit” leukemia/lymphoma, defined by a translocation involving *MYC* in combination with another translocation involving *BCL2*, warrant further study as they are not readily recognized until transformation has occurred and are associated with a poor prognosis [5,6]. Here we describe a patient with an initial diagnosis of FL that transformed to B-ALL. Detailed molecular and cytogenetic characterization of the transformed specimen using karyotype, fluorescence in situ hybridization (FISH), microarray and gene rearrangement analyses revealed the complex nature of the abnormalities present in this disease. These findings included a karyotype with 84-86 chromosomes, principally of whole-chromosome/whole-arm abnormalities, both *IGH*/*BCL2* and *MYC*/*IGH* fusions involving the same single *IGH* allele, as well as limited single-gene alterations involving *NEIL1*, *GPNH*, and *EP300*.

### Case presentation

A 52-year-old man presented with early satiety, fevers and myalgias. His past medical history was remarkable for recent recurrent methicillin- resistant *Staphylococcus aureus* (MRSA) cellulitis. On biopsy, an inguinal lymph node was involved with back-to-back lymphoid follicles containing cells with vesicular chromatin and prominent nucleoli. The cells of interest were CD45+, CD20+, CD10+, BCL2+, BCL6+ and kappa restricted; ki-67 stained 30-40% of the cells. A diagnosis of follicular lymphoma was made and staging studies revealed stage 3 disease, with supraclavicular, precarinal, mediastinal, periaortic, iliac and inguinal adenopathy, and splenomegaly. Lactate dehydrogenase (LDH) was modestly elevated. R-CHOP therapy was initiated with two cycles given 32 days apart. The patient’s symptoms resolved and he did not receive additional therapy. PET/CT imaging done 8 weeks and 6 months after cycle 2 showed resolution of adenopathy, splenomegaly and hypermetabolic activity.

Six months later the patient re-presented with early satiety, easy bruising and fatigue. His CBC showed WBC 7,000/ul with 20% circulating blasts, Hemoglobin 6.6 g/dL and platelet 9,000/ul. LDH was within normal limits. A PET/CT revealed enlarged hypermetabolic lymph nodes in the neck and chest. Splenomegaly was again seen. A bone marrow aspirate and biopsy showed >95% cellularity; 65% of the cells were lymphoblasts with CD10+ CD19+ CD38+ CD58+ CD71+ HLA DR+CD79+ and dim surface lambda positivity. CD20 and kappa were negative. Fine needle aspiration of a supraclavicular lymph node yielded a population of lymphoblasts with the same immunophenotype as the marrow infiltrating cells. A diagnosis of B-cell ALL was made. The patient was treated with 8 cycles of hyperCVAD/MTX-AraC, enjoyed a brief remission, but subsequently relapsed. He refused additional aggressive care and died shortly after relapse.

### Molecular and cytogenetic studies

**Figure 1:**
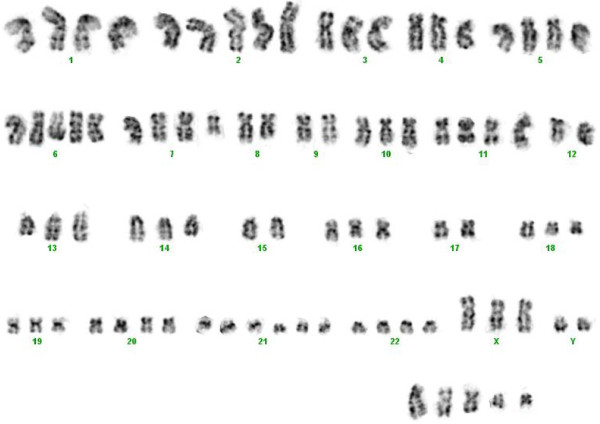
**A representative karyotype with GTG banding.** 84<3n>, XXY,+X,+Y,+1,+2,+2,+5, +del(6)(q13)x2,+del(7)(q11.2),-8,-9,+11,-12, add(13)(q31)x2,-15,-17,del(18)(q21.1),+20,+21,+21, +21,+22,+5mar.

Cytogenetic analysis of the transformed specimen showed a complex karyotype with cells containing 84-86 chromosomes. A representative karyotype is shown in Figure 1. Due to the complexity of the abnormalities it was not possible to discern the presence of any specific translocation by karyotype. Initial FISH analysis, requested due to patient’s history of FL, revealed an atypical *IGH*/*BCL2* fusion signal pattern in 75% of the interphase cells.

Two independent oligonucleotide-based comparative genomic hybridization (CGH) analyses were performed using DNA extracted from the bone marrow specimen obtained for cytogenetic evaluation at the time of diagnosis of B-ALL. These analyses included traditional array CGH (aCGH) for the detection of copy number alterations and a modification of that technology, translocation CGH (tCGH), designed to detect a set of balanced translocations in a single multiplexed assay. Each CGH assay employed the same microarray platform (Signature OncoChip™ manufactured by Roche NimbleGen, Madison, WI) as described in our previous studies [7-9]. For tCGH, unique DNA primers with an average spacing of one primer every 2,000–3,500 bp were designed to linearly amplify genomic regions at recurrent translocation breakpoints for the specific genes involved. Primers were assembled into two reaction pools applied to independent arrays (Pool 1=*BCL11A*, *BCL2*, *CCND3*, *MYC* and *PAX5* with 36, 14, 33, 4 and 15 primers, respectively; Pool 2=*IGH*, *IGK*, and *IGL* with 19, 50 and 20 primers, respectively [10] and http://www.signaturegenomics.com/oncochip_B-cell_ALL.html; OncoChip™ | B-ALL Panel 2). Linear amplification reactions were performed using the Failsafe PCR System with Premix Choice using a single buffer (Premix D, Epicentre, Madison, WI, USA), 200 nM for each primer, and 600 ng genomic DNA. Amplified DNA and unamplified genomic control DNA were labeled with independent Cyanine dyes Cy5 and Cy3, respectively using the NimbleGen Dual-Color DNA Labeling Kit (Roche NimbleGen), co-precipitated and hybridized to the arrays and washed as recommended by the manufacturer. Results were displayed and analyzed with Oncoglyphix® data analysis and visualization software (Signature Genomics, Perkin Elmer, Inc., Spokane, WA). aCGH results were consistent with a hyperdiploid karyotype. Note that aCGH cannot discern ploidy. Based on the karyotype findings, normal microarray results appear to reflect a triploid (3n) genotype. Consistent aberrations detected by both karyotype (presented as 3n) and aCGH included whole chromosome gains for chromosomes 1, 2, 5, 11, 20, 21, 22, X, and Y; partial gains for 6p, 7p, and 13q; as well as losses and partial losses for chromosomes 8, 9, 12, 15, 17, and 18q (Table 1).

**Table 1:** Karyotype and microarray findings in patient

**Karyotype**	**Clinically significant aCGH findings**	**Gene-specific alterations (<1 Mb)**
84-86<3n>,XXY,+X,+Y,+1,+2,+2,+5, +del(6)(q13)x2,+del(7)(q11.2),-8,-9, +11,-12, add(13)(q31)x2,-15,-17, del(18)(q21.1),+20,+21,+21,+21, +22,+4-6mar[cp10]	arr Xp22.33(1-20,008,989)x2,Xp22.12q28(20,008,989-154,876,029)x3,Yp11.32q12(110,058-57,735,230)x2,1p36.33q21.1(356,951-145,048,953)x3,1q21.1q44(145,048,953-246,504,007)x4,2p25.3p11.2(44,198-88,771,193)x2~3,2p11.2(88,771,193-88,939,959)x0~1,2q11.1q12.2(95,004,544-105,862,051)x4,2q12.2q37.3(105,862,051-242,951,149)x2~3,3q25.32q26.31(159,179,678-176,119,209)x4,3q26.31q29(176,182,216-194,089,843)x1,3q29(194,144,086-199,227,915)x2~3,5p15.33q23.1(129,331-120,819,561)x3,5q23.1q23.3(120,866,799-129,327,006)x1,5q23.3q35.3(129,369,711-180,619,169)x3,6p25.3p12.1(128,203-56,479,183)x4,7p22.3p11.1(130,978-57,496,580)x2~3,8p23.3q22.1(177,781-97,839,693)x1,8q24.21q24.3(128,820,805-146,263,042)x2~3,9q12q34.3(69,466,291-140,130,559)x1,11p15.5q25(188,204-134,425,038)x2~3,12q12q21.31(39,127,058-82,868,278)x3,12q24.11q24.12(108,763,306-110,348,984)x1,13q12.11q14.11(18,454,945-40,050,795)x3,13q14.11q21.31(40,056,371-63,246,713)x1,13q31.2q31.3(87,649,097-92,584,929)x5,14q23.3(66,039,131-66,735,897)x3,14q32.33(105,133,180-106,340,244)x1,15q24.2(73,418,043-73,657,137)x1,17p13.3p13.2(49,128-4,959,848)x1,17p13.2p13.1(5,175,207-8,069,489)x1,17p13.1q25.3(8,094,106-78,612,915)x3,18q21.33q23(58,915,605-76,100,854)x1,20p13q13.33(16,653-62,359,694)x3,21q11.2q22.3(14,406,100-46,915,771)x3,22q11.1q11.22(15,912,798-20,879,638)x3,22q11.22(21,356,310-21,571,621)x1,22q11.22q13.2(21,593,777-39,690,567)x3,22q13.2(39,690,567-39,876,284)x>4,22q13.2q13.33(39,876,284-49,691,432)x3	Deletion
		NEIL1 (15q24.2)
		Copy Gain
		GPHN (14q22.3)
		Amplification
		EP300 (22q13.2)

**Figure 2:**
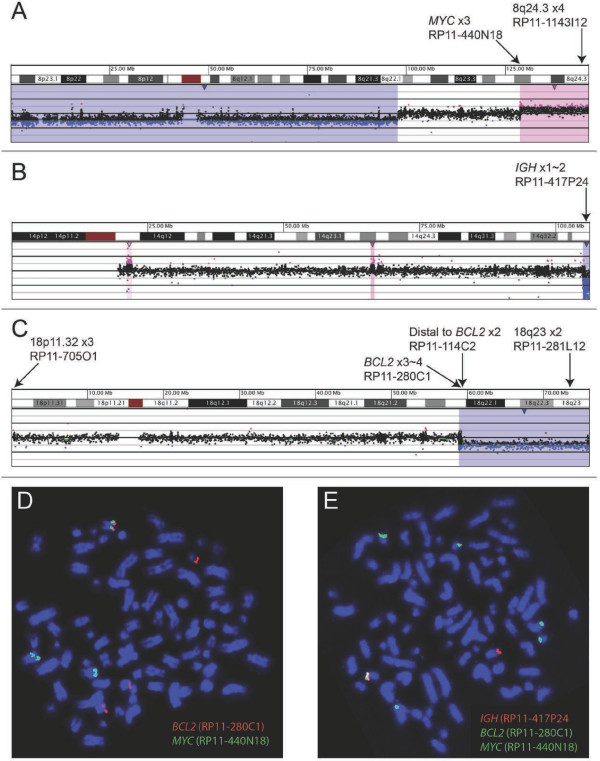
**Microarray and FISH findings of transformed follicular lymphoma.** Copy losses (blue) and copy gains (pink) for oligonucleotide array-based cooperative genomic hybridization (aCGH) are shown for chromosomes **(A)** 8, **(B)** 14, and **(C)** 18. Note that the zero value for Log2 ratio is reflective of a triploid (3n) genotype (confirmed by karyotypes and FISH), as aCGH cannot discern autosomal ploidy. FISH using indicated BAC probes illustrate **(D)** co-localization of *MYC* and *BCL2* on a marker chromosome, and **(E)** co-localization of these genes with *IGH* on the same marker. Probe colors match the indicated probe names.

**Figure 3:**
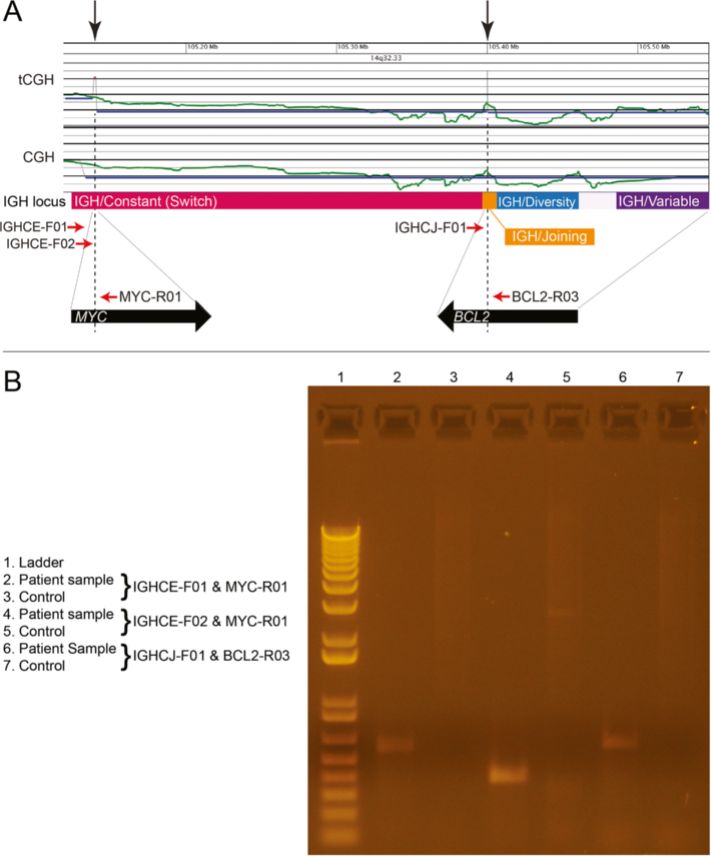
**Molecular delineation of complex rearrangement of the marker chromosome bearing *MYC*, *IGH *and *BCL2*. (A)** OncoChip™ | B-ALL Panel 2 and standard aCGH are shown for comparison with results indicative of two rearrangements (black arrows on top), one corresponding to the Constant region (class switch) and the other corresponding to the Joining region of the *IGH* locus as indicated. Locations of primers designed for subsequent confirmatory PCR reactions (red arrows), and the location and directionality of the *MYC* and *BCL2* genes are also indicated. The lines originated from *MYC* and *BCL2* indicate that *MYC* is inserted into the *IGH*, while *IGH/BCL2* fusion is resulted from a translocation. **(B)** PCR reactions using the indicated primers confirm the insertion of *MYC* gene into *IGH* Constant region and translocation of *BCL2* gene to the *IGH* Joining site.

**Figure 4:**
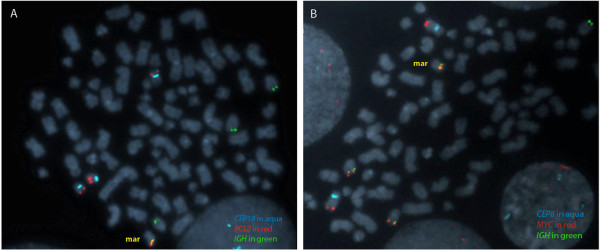
**FISH confirmation of a double-hit rearrangement. (A)***IGH/BCL2* fusion and **(B)***MYC/IGH* fusion are present on a marker chromosome. Centromeres of chromosomes 8 and 18 were shown in aqua as indicated. Images of Figures 2E and 3A demonstrated that the fusions were on the same marker chromosome.

Gene-specific findings included a gain of the distal long arm of chromosome 8 with a breakpoint near the distal 5’ end of *MYC*, and losses of the terminal long arms of chromosomes 14 and 18 with breakpoints within the *IGH* locus and just proximal to *BCL2* gene, respectively (Figure 2). aCGH revealed relatively few gene-specific alterations that included deletion of *NEIL1* (15q24.2), mosaic single copy gain of *GPHN* (14q22.3) and amplification of a truncated *EP300* allele (22q13.2) (Table 1). OncoChip™ | B-ALL Panel 2 analysis identified the *IGH*/*BCL2* fusion as well as a *MYC*/*IGH* rearrangement (Figure 3A), which were subsequently confirmed by FISH and PCR (Figures 2D and 2E, and Figure 3B, respectively). The *MYC/IGH/BCL2* complex rearrangement appeared to be on a marker chromosome, which was further confirmed by using the commercially available dual fusion FISH probes (Abbott, Abbott Park, IL) spanning the translocation breakpoints (Figure 4).

## Discussion

Follicular lymphoma transforming into acute lymphoblastic leukemia has been documented in a limited number of cases [2,3,11,12]. “Double-hit” B-cell malignancies with both *BCL2* and *MYC* rearrangements have also been reported in de novo large B-cell lymphoma [13] and Burkitt’s lymphoma/leukemia with three-way translocation t(8;14,18) [14]. Such “Double-hit” B-cell malignancies are characterized by an extremely aggressive course and a high relapse rate [5,6,13,14]. Given the initial diagnosis of follicular lymphoma in our case, *IGH/BCL2* fusion was likely the first event, with *MYC/IGH* fusion arising secondarily and accompanying a complex karyotype. An early specimen at the time of diagnosis of FL was not available for this case.

Double-hit translocations in leukemia or lymphoma rarely occur at a single immunoglobulin locus. Knezevich et al. reported the detection of a complex der(8)t(8;14;18) in a few cases with a *MYC/IGH/BCL2* rearrangement resulting from translocation of *BCL2* and *MYC* involving a single disrupted *IGH* allele [15]. Molecular cytogenetic investigation is essential to identify and characterize such cases. In our case the *IGH* breakpoints for the *BCL2* and *MYC* rearrangements were located within the switch region ([hg18] Chr14:105140803-105142224) and at the start of the joining region ([hg18] Chr14:105400386-105400678), respectively, which is consistent with the previous reports [16]. The *MYC* breakpoint was located within intron 1 ([hg18] Chr8:128818114-128818314), which is also a recurrent site. Most cases of sporadic Burkitt lymphoma and B-ALL exhibit *MYC* breakpoints either in the first intron, the first exon, or upstream of the gene [16]. In contrast, the breakpoint for *BCL2* was detected at the 5’ end of the gene ([hg18] Chr18:59135900-59136028). This is atypical, as *BCL2* rearrangements predominantly involve defined major and minor breakpoint regions, both of which are 3’ of the gene [16]. 5’- *BCL2* breakpoints have been described, but these are rare and nearly always involve the immunoglobulin light chain, not seen in this case.

In addition to the *MYC/IGH/BCL2* rearrangement, microarray analysis detected copy number alterations in this case. Although the majority of the alterations were large and involved whole chromosomes or whole arm loss or gain, a few smaller alterations, including deletion of *NEIL1*, mosaic single copy gain of *GPHN,* and amplification of a truncated *EP300* allele were identified. *NEIL1* is a member of the Nei endonuclease VIII-like gene family which encodes DNA glycosylases. The *NEIL1* encoded enzyme catalyzes the removal of specific oxidative damage from DNA and interacts with key factors in cell cycle checkpoints and repair. *NEIL1* is among the genes for which expression levels have been used to distinguish subtypes of diffuse large B cell lymphoma [17]. *GPHN* (for gephyrin, from the Greek word for “bridge”) is a neuronal receptor assembly protein that links membrane-associated receptor molecules to cytoskeletal microfilaments. Interestingly, *GPHN* has been shown as a fusion partner of *MLL* in leukemia, and the *MLL-GPHN* fusion gene can transform hematopoietic progenitors [18]. *GPHN* has also been found to be upregulated by a B-cell- activating factor as part of a cascade of events that inhibit CD20-mediated and B-cell receptor-mediated apoptosis in human B cells [19]. *EP300* encodes the adenovirus *E1A*-associated cellular p300 transcriptional co-activator protein. It functions as a histone acetyltransferase that regulates transcription via chromatin remodeling and is important in the processes of cell proliferation and differentiation. Mutations in *EP300* and the highly related *CREBBP* are the most frequent structural abnormalities in follicular lymphoma and diffuse large B-cell lymphoma [20,21]. In addition, acquisition of *EP300* mutation has recently been shown in the progression of severe congenital neutropenia to acute myeloid leukemia [22]. The findings of these additional aberrations may represent events facilitating the transition of FL to B-ALL. The results presented in this study identified genomic alterations with potential relevance to transformation and emphasize the value of employing diverse cytogenetic techniques in the workup of cases of leukemia and lymphoma.

### Consent

Written informed consent was obtained for publication of this case report. A copy of the written consent is available for review by the Editor-in-Chief of this journal.

## Competing interests

The authors declare that they have no competing interests.

## Authors’ contributions

YN supervised cytogenetic study and performed FISH analysis. AF, NN, and TM and RS performed microarray assay, analyzed data, and provided interpretation. ASK provided clinical information and obtained consent. RS and YN wrote this report. All of the authors contributed to finalize and approve the final version of this manuscript.
